# Content and Feature Preferences for a Physical Activity App for Adults With Physical Disabilities: Focus Group Study

**DOI:** 10.2196/15019

**Published:** 2019-10-11

**Authors:** Sara H Olsen, Sandra L Saperstein, Robert S Gold

**Affiliations:** 1 Department of Behavioral and Community Health School of Public Health University of Maryland College Park, MD United States

**Keywords:** adapted sport, disability, physical activity, focus group, apps, fitness, user-centered design, mHealth

## Abstract

**Background:**

Hundreds of thousands of mobile phone apps intended to improve health and fitness are available for download across platforms and operating systems; however, few have been designed with people with physical disabilities in mind, ignoring a large population that may benefit from an effective tool to increase physical activity.

**Objective:**

This study represents the first phase in the development process of a fitness tracking app for people with physical disabilities interested in nontraditional sport. The aim of this research was to explore user preferences for content, appearance, and operational features of a proposed physical activity app for people with physical disabilities to inform the design of a mobile phone app for increasing physical activity.

**Methods:**

Four focus groups were conducted with 15 adults with physical disabilities who currently participate in nontraditional, non-Paralympic sport. Data collected from the focus group sessions centered on content, functionality, and appearance of apps currently used by participants as well as preferences for a future app.

**Results:**

Participants (mean age 35.7, SD 9.2 years) were mostly white (13/15, 87%), and all were currently participating in CrossFit and at least one other sport. Five main themes were identified. Themes included preferences for (1) workout-specific features that were tailored or searchable by disability, (2) user experience that was intuitive and accessible, (3) profile personalization options, (4) gamification features that allowed for competition with self and other users, and (5) social features that allowed increased interaction among users. Participants expressed a primary interest in having a fitness app that was designed for people with physical disabilities such that the features present in other fitness tracking apps were relevant to them and their community of adaptive athletes.

**Conclusions:**

The results showed that features related to user experience, social engagement, and gamification are considered important to people with physical disabilities. Features highlighted by participants as most desired, from a consumer perspective, were in line with research identifying attributes of quality apps that use behavior change techniques to influence positive physical activity behavior change. Such insights should inform the development of any fitness app designed to integrate users with disabilities as a primary user base.

## Introduction

### Background

The physical, psychological, emotional, and economic benefits of participating in physical activity are well established. Regular, moderate-intensity physical activity has been shown to improve muscular and cardiorespiratory fitness as well as bone health and reduce the risk of chronic diseases such as hypertension, coronary heart disease, diabetes, various cancers, and depression. Physical activity plays a critical role in weight control, which has also been linked to the same chronic diseases [1-4]. Conversely, physical inactivity has been attributed to 6% of coronary heart disease, 7% of type 2 diabetes, 10% of breast cancer, and 10% of colon cancer incidence globally [5]. Research also suggests that even small increases in physical activity in the least active populations have a much larger impact on overall health than any increase in activity level in populations closer to achieving physical activity guidelines [5]. These findings provide a powerful rationale for interventions to increase physical activity.

The Centers for Disease Control and Prevention broadly defines disability in adults as a limitation to routine activities or the need for assistive devices. More than half of the adult US population with any disability is inactive—47% are completely sedentary and an additional 22% do not move enough to meet the physical activity guidelines [6], which is higher than the national sedentary estimate of 23.7% in the general population [7]. This study focuses on people with physical disabilities, that is, those individuals with permanent physical impairments that affect participation in physical activity and fitness. People with physical disabilities, on the whole, are more likely to report poor overall health, lower access to health care, and decreased physical activity [8] than those without disabilities. This population is at significantly greater risk of comorbidities associated with inactivity [9,10], with risk of disease increased regardless of disability type [7]. Scoping reviews of literature report the use of mobile health (mHealth) to improve physical activity in special populations [11,12], with 1 randomized controlled trial showing significant increases in physical activity over time in internet-based physical activity interventions with adults with multiple sclerosis [13]. However, the research for people with physical disabilities is limited. Expansion of electronic health (eHealth) approaches to increase physical activity should continue to be investigated for all people with physical disabilities, with a focus on mobile apps as the market continues to expand.

There are 380,000 mobile apps related to health and fitness, with over 78,000 added in 2017 alone [14]. mHealth apps focusing on physical activity tend to lack theory or evidence-based content and messaging, and few of the commercially available apps have been actively studied for their effectiveness to increase physical activity or intent to continue activity [15,16]. However, there is evidence that eHealth interventions (internet-based and native mobile apps) that target physical activity resulted in increases in users’ activity [17,18], and mobile physical activity app users are more likely to maintain behaviors than nonusers [19]. In addition, taking advantage of gamification features in mHealth apps resulted in improved user health and well-being [20].

Increased access to technology, specifically mobile apps, may be able to decrease prevalence of inactivity of users, including users with physical disabilities. Plow and Golding [21] found that the use of commercially available apps designed to promote self-management showed increases in planned physical activity in adults with musculoskeletal and neurological conditions. People with musculoskeletal conditions also adhered better to home therapy exercise routines and were more likely to increase function when prescribed programs were available on an app, compared with take-home paper instructions [22]. App use shows promise for people with physical disabilities, but apps are not generally designed specifically for these users, causing accessibility issues during use. Mobile devices’ small screens with little negative space make using the device difficult for people with visual impairments or dexterity issues [23]. Therefore, people with physical disabilities have to adapt to the app rather than have an app that is intuitive for them. Active people with physical disabilities understand the unique physical activity needs of people with physical disabilities and have the experience to inform design of an app that is more likely to meet their needs at various levels of physical activity participation.

### Expanding Landscape of Adapted Sport

In the United States, traditional recreational sport competitions have had to make room for nontraditional activities, such as functional fitness, obstacle course racing (OCR), and strongman competitions. Marathons and shorter races have seen registrations on a slow decline after peak registrations in 2013 [24]. Meanwhile, there is a growth in participation in less traditional OCR. Events such as the Spartan and Tough Mudder report 5 million and 2 million participants, respectively [24]. CrossFit, another nontraditional sport, has risen globally for the last 10 years. By the beginning of 2019, there were approximately 15,500 registered affiliates worldwide [25].

Parasport activities, sports such as wheelchair basketball and track events that are represented in Paralympic competition, are promoted through local parasports clubs, the Department of Veterans Affairs, and a variety of adapted sport-based nonprofit organizations. In recent years, people with physical disabilities have increased their recreational participation in nontraditional sports and are being recognized by organizers as consumers of nontraditional sport. In 2018, Spartan introduced the first ever Para Spartan Heats. Adaptive athletes (also known as people with physical disabilities) in the Elite Para division competed for a grand prize of US $10,000, and the event was covered by the Entertainment and Sports Programming Network, a major sports-broadcasting network. Although anecdotal, the expansion and visibility of people with physical disabilities in large nontraditional sport events is indicative of the growing interest of the community. As interest continues to grow, so might the consumer market for physical activity apps for them. To maximize the potential for proposed apps to support behavior change, specifically sustained physical activity participation, the apps should be designed to meet the needs and desires of the target audience, the people with physical disabilities, and should use evidence-based design features.

### User-Centered Design

From a commercial perspective, investment in design and development of an app is paramount because if a consumer is not engaged with the app, they will not return or recommend it to others. This also applies to mHealth—if the end user is not engaged, the intervention effectiveness is undermined [26]. Understanding the end-user requirements and the context of use increases a design’s success rate of usability and, ultimately, the engagement necessary for an mHealth intervention implementation [27]. Currently, however, commercial apps do not integrate health intervention best practices, and mHealth interventions do not include the end user in the design process, reducing the potential effectiveness of the app to create sustained behavior change [26].

Focus groups can be a valuable tool in the early stages of the user-centered design (UCD) process to better understand what users want in the app. For a new app with broad potential reach, the type and number of actual users may be unclear until launch. Therefore, defining primary user characteristics is necessarily an iterative process [28]. This study recruited currently active people with physical disabilities who had used a fitness tracking app at least once to understand the unique needs of people with physical disabilities and to begin to build a taxonomy of features for reference in future focus groups of people with physical disabilities who might be less familiar with fitness tracking apps.

### Objective

The indication that mobile app use increases physical activity adherence and the growth of participation of people with physical disabilities in nontraditional sport emphasizes the potential for mobile fitness tracking apps, designed specifically for users with physical disabilities, across a wide variety of physical activities to influence positive health outcomes. This study represents the first phase of UCD data collection for the development of an app for people with physical disabilities. The aim of this study was to explore user preferences for content, appearance, and operational features for a physical activity app for people with physical disabilities. Findings from this study will be used to inform the initial design phase of the mobile app.

## Methods

### Study Design

To encourage interaction and generation of ideas related to a proposed fitness tracking app for people with physical disabilities, focus groups were chosen. Focus groups offer an opportunity for discussion, debate, and idea sharing that has the potential to draw out more nuanced preferences of app content and features [29]. A total of 4 focus groups comprising adults with physical disabilities who were active in nontraditional sport (eg, no Paralympic equivalent) were conducted in the south, west, and mid-Atlantic regions of the United States throughout the months of March and April 2018. Focus groups were conducted by an experienced facilitator and an observer note taker. The facilitator guide was reviewed by a professional focus group moderator who made suggestions to improve wording and flow of questions. Focus group sizes averaged 4 participants. Institutional review board approval for data collection with active consent was obtained in advance through the University of Maryland.

### Participants

Adults, aged 18 to 54 years (mean age 35.7, SD 9.2 years), who had a self-reported physical disability and were currently engaged in nontraditional sport activities were recruited through Crossroads Adaptive Athletic Alliance’s network of fitness venues and via internet-based posting in closed Facebook groups built for adaptive athletes. A total of 15 people volunteered to participate. Participants were mostly white (n=13) and approximately half of all participants were female (n=9 female). Patients varied in mechanism of disability (n=8 acquired, n=6 congenital, n=1 both). The most prevalent nontraditional sport listed was CrossFit (n=15), and all participants engaged in at least one other sport. The demographic data, as collected on a questionnaire, for participants are presented in Table 1.

**Table 1:** Demographic characteristics of focus group participants across 4 focus group sessions and 3 regions in the United States.

Demographic characteristics		Focus groups participants (N=15), n (%)
**Gender**		
	Female	9 (60)
	Male	6 (40)
**Age (years)**		
	18-29	3 (20)
	30-39	7 (47)
	40-49	4 (27)
	50-59	1 (7)
**Race or ethnicity**		
	White	13 (87)
	African American	1 (7)
	Asian	1 (7)
**Region**		
	South	8 (53)
	West	4 (27)
	Mid-Atlantic	3 (20)
**Disability**		
	Lower extremity amputation	4 (27)
	Cerebral palsy	3 (20)
	Spinal cord injury	3 (20)
	Other (eg, club foot and limb paralysis)	3 (20)
	Upper extremity amputation	1 (7)
	Visual impairment	1 (7)
**Mechanism**		
	Acquired	8 (53)
	Congenital	6 (40)
	Both	1 (7)
**Current sport participation**		
	CrossFit	15 (100)
	Parasport (ie, Paralympic equivalent available)	7 (47)
	Olympic lifting	4 (27)
	Baseball	1 (7)
	Bodybuilding	1 (7)
	Highland games	1 (7)
	Obstacle course racing	1 (7)
	Sailing	1 (7)
	Strongman	1 (7)
	Volleyball	1 (7)
	Yoga	1 (7)

### Procedures

Before the sessions began, participants were informed about the purpose of the groups, what they would be asked, the benefits and risks of participation, that participation was voluntary, and how their responses would be kept confidential. Participants signed and received a copy of the informed consent forms. Focus group sessions lasted approximately 2 hours and were audio recorded. Lunch was provided during the focus group sessions, but no other incentives were offered.

A semistructured guide was developed to frame the discussion. The session was designed to cover 4 broad topic areas. The first part of the session examined participants’ introduction to adapted sport. The second part explored current app use. Participants were asked to describe the apps they used most frequently and what features they liked most. The question was used to elicit features that appealed to participants, regardless of app purpose. All participants were current app users with the most common apps mentioned being games or social media. However, apps organically introduced by participants also included fitness tracking apps. Regardless of app type, preferred features that were mentioned by the participants were included in the final ranking activity. In the third phase of the focus groups, participants were provided screenshots from 2 mobile apps: Gateway to Gold, designed by US Paralympics as a fitness tracking and recruiting tool specifically for people with physical disabilities, and Beyond the Whiteboard, the official app of CrossFit to track workouts and connect a geographically disparate fitness community. None of the participants had used either app, although a few mentioned that they had heard of Beyond the Whiteboard. On the basis of images alone, participants provided feedback and described functionality assumptions. In the final phase of the focus groups, participants were asked to consider their own experiences and the session’s discussion to describe the features of an ideal fitness tracking app for people with physical disabilities. Some of the gamification and social interaction features participants described in this phase of the focus group were originally mentioned in the second phase of the focus group in which features of most frequently used apps were discussed. The end of each session included an activity in which app features mentioned by the participants throughout the session were displayed for participant review. Therefore, each focus group had a slightly different list of features to review. Table 2 lists these features such as offering broad disability selection options for user profiles. The participants independently rated priority features as *must have*, *nice to have*, or *not needed*. They were not required to vote on every feature.

**Table 2:** Focus group participants’ identification and rankings of preferred features in a disability-specific workout app.

Theme and identified features^a^		“Must have”	“Nice to have”	“Not needed”	Total participants rating this feature
**Profile**					
	Broad profile classifications of disability (vs more traditional narrow Paralympic classifications)	2	—	—	11
	Customizable profile (avatar, location, social media handles, etc)	1	1	—	6
	Subcategories of disability category available for selection on profile page	—	—	—	9
**Workout-specific**					
	Detailed and strict movement standards for multiple disabilities for each posted workout	8	—	—	15
	“Additional assistance needed”/modification options for workouts	8	—	—	15
	Workout history (view by workout or calendar)	7	—	—	12
	Workout search function (by primary muscle group/body part or specific movement)	2	3	—	8
	Workout/training tips	2	—	—	6
	Wholistic tracking (nutrition, sleep, etc)	—	2	—	13
	Lift percentages precalculated based on 1RM^b^, 3RM, or 5RM in the benchmark section	—	2	1	7
**User experience**					
	Links to an in-app resources page (grants for equipment and travel, used adapted sport gear, link to Parasports club pages, etc)	5	—	—	13
	Intuitive functions and navigation	4	—	—	13
	Compatible with phone screen readers	2	—	—	4
	User control of what others can see, privacy settings	2	—	—	9
	Free	1	—	—	9
	User video upload	1	—	4	9
	Glossary of terms	—	3	—	6
	Explanation on landing page of who the app is for/who is allowed to join	—	—	3	6
**Gamification features**					
	Leaderboard sortable by location, disability, and gender	4	6	—	15
	Goal setting in app	—	4	—	4
	Share PRs^c^ and workout comments in app	—	2	—	5
	In-app prompts to move up a level or try a heavier weight (based on time since last attempt, comparison with others at the same level, or progress marked in other benchmarks)	—	2	—	13
	Levels defined for each workout, with next level locked until previous one is completed	2	—	2	15
	Profile badge for “level” achieved in app	—	3	2	9
	In-app notifications (eg, PR, movement on leaderboard)	—	2	4	9
	Option to make scores public or private	—	—	2	9
**Social/user interaction**					
	Ability to network with and message other users	2	2	—	12
	Share in-app successes on social media	—	3	—	15
	Positive reinforcement from other users (“like” score, post emoji)	—	—	2	7
	Compete with friends in app (eg, post own challenges)	—	—	2	7
	Ability to create workout groups with other users	—	—	1	4
	Ability to look up other athletes	—	—	1	10
	Ability to comment on workout descriptions	—	—	1	6
	Sync results to fitness tracker	—	—	1	6

^a^The numbers in each column reflect aggregated votes across all focus groups. Participants were asked to place colored stickers by features to represent *must have*, *nice to have*, and *not needed* app features but not required to vote on each feature. Some features received no stickers but remained on the table to indicate being mentioned during the focus group session.

^b^RM: repetition maximum, for example, 1RM is the maximum weight lifted by an individual for 1 repetition.

^c^PR: personal record.

### Data Analysis

During the focus groups, notes taken by the facilitator and note taker were compared to ensure all mentioned features were included in the final activity. Participants were also asked to review the list to ensure nothing was missed or presented other than intended during conversations. Saturation was reached by the fourth focus group session. This list formed the basis of the results. In addition, focus group sessions were recorded and transcribed verbatim and verified for accuracy by a second listening. Using the processes inherent in thematic analysis [30], each focus group transcription was open coded by a single researcher (SHO) with the review of thematic analysis by coauthors (SLS and RSG). Codes related to the app features were then collated into potential themes and compared against coded data extracts. The full list of feature themes derived from the transcriptions was used to verify the completeness of the lists used during the focus group rating activity. No additional feature-based themes emerged from the data during this last step.

## Results

### Summary

Analysis of focus groups sessions revealed 5 overarching themes across the 34 recommended features and functions. The themes address preferences for (1) workout-specific features that were tailored to or searchable by disability, (2) user experience that was intuitive and accessible, (4) profile personalization options, (4) gamification features that allowed for competition with self and other users, and (5) social features that allowed increased interaction among users. Several of the user experience and social feature preferences mentioned by the participants were based on apps and app features that were not fitness related. The complete list and total number of votes of each feature aggregated across all focus groups can be found in Table 2.

### Profile Features

Participants noted that the profile setup page for the comparison Gateway to Gold app used very specific Paralympic-based classification terms for the profile (eg, Les Autres and Dysmelia) that participants did not relate to or identify with. Given the fact that the proposed app would not be built specifically for Paralympic hopefuls, participants believed the categories should be less specific and more understandable to all users. They also wanted the ability to personalize their profile page.

Participants mentioned the preference for customizable profile pages including video upload:

…the humor part of it, it's part of expression, personalizing it. That's another thing just being able to personalize something. I don't know that's the girly side of me, if I can make it pink, would I? Or whatever, you know, something to personalize it. Everybody wants it to be their own somehow.

Participant 2

I know sometimes if you're posting videos of what you're doing it might inspire someone else to do that or you'll see it and say, oh I can do that, as opposed to reading it. It makes a different connection.

Participant 10

### Workout-Specific Features

Both comparison app screenshots showed a library of available workouts. In a proposed app that was designed for a wide variety of people with physical disabilities, participants were primarily concerned with understanding how to do a new workout or fitness movement contained in the library. They wanted workouts with links to short videos contained in an in-app library that demonstrated a movement with multiple adaptation options, clearly described standards for proper completion of movements (eg, a squat for a below the knee amputee would mean hips drop below parallel on each repetition) differentiated by broad classifications of athletes, and provided recommendations for modifying the movements if the given standards were too hard for the user. It was also important for the participants to be able to see a history of their workouts to quickly determine improvement and set new goals. The example most frequently given was when a workout was opened from the search page, the dates and scores of all previous attempts would be listed for the user who was logged in.

Participants mentioned the preference for in-app demonstration videos of a wide variety of movements and standards for a wide variety of disability categories (Rx: how a movement or workout is prescribed by the coach or fitness instructor; denotes the weight, movements, and number of repetitions in a workout; used by participants generally referring to workouts similar to CrossFit):

…what I mean by that is there's a difference between him and her. Right? And between me and you. So, what my thought is you could click on your ability level basically and it would have a video or something like that of how you do it. So, it wouldn't just have the one. You know what I'm saying?

Participant 6

Or like a section with videos posted by the app, to show you how to do a movement, kind of like an instructional video from professionals instead.

Participant 10

The exact thing that the Open does specifically. They have the different variations that give a video of the exact workout for the RX athlete. Then you have specific individual photos to show the scaled versions for whoever it is, whatever category and whatever person it is.

Participant 8

### User Experience

Participants spoke of the unique position many adaptive athletes find themselves in as the only person they know attempting sport and how that alone can be a barrier to participation. They recommended a resources page with links to adaptive sport event calendars, popular adaptive devices used in sport, and grant opportunities to enable participation. From a user experience perspective, participants noted the text-heavy nature of both Gateway to Gold and Beyond the Whiteboard apps. The amount of information on each screen was distracting, and they would prefer an app that is more visual and more intuitive. A participant complained:

It does have pretty small text. It's too busy. Too much information that I don't care.

Participant 2

A positive feature noted by a participant with visual impairments was the large text in the buttons and use of contrasting colors in the Gateway to Gold app that made navigation easier for her than in other apps in which she had to rely solely on her screen reader.

The participants explained why a resource page was important to adaptive athletes:

Let's just say in my condition right now, I want to get into wheelchair basketball and if I'm watching other people on this app doing these things. If I'm going, “I know my wheelchair isn't that one; it can't do that,” again there's that resource. Click on this; its highlighted. This is where you get that type of resource.

Participant 2

I know as a blind person I never really heard much about sports or inclusive sports.

Participant 11

### Gamification

Participants stated that in addition to tracking their own progress, they wanted the ability to compare their results with others, and more importantly, others similar to themselves. Several participants used FitBit, Wodify, and MyFitnessPal but were frustrated that wheelchair versus run options or 1-arm lifts were not available selections, making it impossible to compare themselves with others on the app. The most appealing option, therefore, was a leaderboard that would allow them to sort scores by disability type, adding competition with others *like them* to the app and making it unique from all other apps currently used. Participants wanted to be able to sort by disability and click on profiles of other athletes. Additional gamification features mentioned were related to scores in workouts such as personal record notifications that matched features they already used in other apps.

The participants expressed their opinions on the preferred use of an in-app leaderboard:

You compete against people who are, have around the same abilities as you, so it would be nice to see where you fall. I think to be able to toggle between a very specific category and then a really broad category.

Participant 10

It's the competitive piece to see how you stack up against others.

Participant 7

### Social Features

Despite all participants having multiple social media accounts, participants did not prioritize the ability to connect to those accounts or enable cross-posting across apps. Features participants liked in other apps that they thought this app should integrate included the ability to post notes, graphics interchange format files, and images to other people’s accounts or feeds within the app and the ability to interact with each other concerning the workouts. They liked encouragement that a simple emoji on a workout score provided and believed that finding someone with a similar disability doing the same workouts might increase their motivation or performance over time.

## Discussion

### Principal Findings

The results of this study support the need for a physical activity app designed for and with people with physical disabilities. Focus group participants discussed and ranked a wide variety of features and content for a proposed physical activity app for people with physical disabilities. Greatest consensus among participants was achieved for features that would make this app unique to people with physical disabilities or offered mechanisms for goal setting and competition, such as clear and detailed movement examples and standards for a wide variety of disabilities, disability-specific modification or progression options for movements that are currently too difficult for the user, resources page unique to adaptive sport, and a sortable leaderboard that includes a filter for disability category.

The features listed as most desirable by participants mirror those found to, empirically, support behavior change. Scoping studies reveal that apps do not intentionally incorporate health behavior constructs [31] nor do they score well on instruments designed to evaluate inclusion of behavior change theory strategies [32]. Because apps on the market do not effectively integrate constructs or strategies of behavior change, consumers may need to use multiple fitness apps to affect behavior change [33]. The inclusion of behavior change techniques, derived from multiple theories and incorporated into a taxonomy for use [34], has been shown to improve the likelihood of change and the maintenance of the newly acquired behavior [33,35,36]. These focus groups, although not explicitly discussing theoretically based development, identified features and content most likely to effectively support behavior change.

The most common and effective behavior change techniques incorporated into commercially available physical activity apps were found in goal setting, behavior feedback, and behavior demonstration features [16,36-38]. Focus group participants listed the ability to comment on other users’ workout results and workout videos, the ability to record and track progress, and the need for a wide variety of demonstration videos as priorities for their ideal fitness tracking app. They also commented frequently about the opportunity this app would give to be a part of a larger, connected adapted sport community and how that connectedness would offer a much wider set of examples for modifications to fitness movements based on disability, something that is not readily accessible to them now. Networkability—the degree to which the app supports a social networking function—has also shown increases in intention to continue using fitness tracking apps [39]. It is clear from the focus group discussions that people with physical disabilities believe a fitness tracking app designed for them would increase the sense of belonging, access to role modeling behavior either vicariously or through in-app interaction, and awareness of adaptive sport participation options.

Commercially available fitness apps incorporate gamification design principles to increase *relatedness* by integrating features that enable users to interact [40]. These same features—leaderboards and messaging and commenting on other users’ posts or successes—are among the features focus group participants most desired. The application of gamification in health and fitness apps suggests that it can lead to a wide variety of positive behavior change impacts [20]. Gamification, specifically the use of leaderboards, has also been shown to significantly influence physical activity participation [41]. Developers marketing to people with physical disabilities should combine behavior change, technical, and design features to encourage increases in physical activity [37]. It is important to take into account unique desires of people with physical disabilities in the design process. Fitness tracking apps for people with physical disabilities should include demonstrations and clear instructions on how to perform suggested movements based on impairment. The user interface for people with physical disabilities, especially those with visual and dexterity impairments, should include high-contrast views with minimal text and large buttons for navigating the app.

### Limitations

This study used a nonrandom purposive sample, collecting data through a network of fitness venues associated with a single nonprofit organization. This recruitment method may have introduced a selection bias; individuals who agreed to participate were already active in nontraditional sport, so their app needs might be different from a person with a physical disability just becoming active. One example of this was the extensive discussion of peer support social features during the focus groups sessions but the low rating of those features in the final activity. For active people with physical disabilities, peer modeling and virtual social support may be less important to sustained physical activity, especially if such support is provided through their sport participation. This may be enhanced by participation in traditional parasport team activities by half of the participants. All participants were active in CrossFit in addition to other sports. The average CrossFit athlete is of high socioeconomic status, whereas the average person with a physical disability is of lower socioeconomic status, which may have influenced the preference of certain features or content. Although the participant group was more homogenous with respect to race/ethnicity, the sample represented people with a wide range of disabilities, sporting interests, and an equitable number of males and females. Only 4 focus groups were conducted; however, data saturation was reached by the fourth focus group, and findings represented a diverse set of participant preferences [42]. Despite saturation of suggested features, the heterogeneity of participant needs was clear in the rating of app features. Only 2 features were considered *must have* by more than half of the participants.

### Conclusions

UCD considers the needs and preferences of the end user at all stages of development. This is particularly important for anyone developing an app for people with physical disabilities; unless you have the lived experience of accessing apps as a person with physical disabilities, designing for this group is challenging. Besides providing input for a more usable platform, the process of using UCD for apps intended for people with physical disabilities empowers an often marginalized population to contribute to their own health. The results showed that features related to user experience, social engagement, and gamification are considered important to people with physical disabilities. Though none of the apps used as examples by the participants integrated theory, the features they highlighted as most desired were in line with the research-identifying attributes of quality apps that used behavior change techniques to influence positive physical activity behavior change. Such insights should inform the development of any fitness app designed to integrate users with disabilities as a primary user base. The heterogeneity of preferences when asked to rate each feature indicates that future research is needed to understand how disability type and physical activity participation affect app feature and content preference of the people with physical disabilities.

## Abbreviations

eHealth: electronic health
mHealth: mobile health
OCR: obstacle course race
RM: repetition maximum
PR: personal record
UCD: user-centered design
